# Advancing healthcare with artificial intelligence: diagnostic accuracy of machine learning algorithm in diagnosis of diabetic retinopathy in the Brazilian population

**DOI:** 10.1186/s13098-024-01447-0

**Published:** 2024-08-29

**Authors:** Mateus A. dos Reis, Cristiano A. Künas, Thiago da Silva Araújo, Josiane Schneiders, Pietro B. de Azevedo, Luis F. Nakayama, Dimitris R. V. Rados, Roberto N. Umpierre, Otávio Berwanger, Daniel Lavinsky, Fernando K. Malerbi, Philippe O. A. Navaux, Beatriz D. Schaan

**Affiliations:** 1 Graduate Program in Medical Sciences: Endocrinology, Universidade Federal do Rio Grande do Sul, Porto Alegre, RS Brazil; 2 Universidade Feevale, Novo Hamburgo, RS Brazil; 3 Institute of Informatics, Universidade Federal do Rio Grande do Sul, Porto Alegre, RS Brazil; 4 Department of Ophthalmology and Visual Sciences, Universidade Federal de São Paulo, São Paulo, Brazil; 5 Laboratory for Computational Physiology, Institute for Medical Engineering and Science, Massachusetts Institute of Technology, Cambridge, MA USA; 6 TelessaúdeRS Project, Universidade Federal do Rio Grande do Sul, Porto Alegre, RS Brazil; 7 Department of Social Medicine, Universidade Federal do Rio Grande do Sul, Porto Alegre, Brazil; 8 The George Institute for Global Health, Imperial College London, London, UK; 9 Department of Ophthalmology, Universidade Federal do Rio Grande do Sul, Porto Alegre, Brazil; 10 Institute for Health Technology Assessment (IATS) – CNPq, Porto Alegre, Brazil; 11 Endocrinology Unit, Hospital de Clínicas de Porto Alegre, Porto Alegre, RS Brazil

**Keywords:** Diabetic retinopathy, Artificial intelligence, diabetes mellitus

## Abstract

**Background:**

In healthcare systems in general, access to diabetic retinopathy (DR) screening is limited. Artificial intelligence has the potential to increase care delivery. Therefore, we trained and evaluated the diagnostic accuracy of a machine learning algorithm for automated detection of DR.

**Methods:**

We included color fundus photographs from individuals from 4 databases (primary and specialized care settings), excluding uninterpretable images. The datasets consist of images from Brazilian patients, which differs from previous work. This modification allows for a more tailored application of the model to Brazilian patients, ensuring that the nuances and characteristics of this specific population are adequately captured. The sample was fractionated in training (70%) and testing (30%) samples. A convolutional neural network was trained for image classification. The reference test was the combined decision from three ophthalmologists. The sensitivity, specificity, and area under the ROC curve of the algorithm for detecting referable DR (moderate non-proliferative DR; severe non-proliferative DR; proliferative DR and/or clinically significant macular edema) were estimated.

**Results:**

A total of 15,816 images (4590 patients) were included. The overall prevalence of any degree of DR was 26.5%. Compared with human evaluators (manual method of diagnosing DR performed by an ophthalmologist), the deep learning algorithm achieved an area under the ROC curve of 0.98 (95% CI 0.97–0.98), with a specificity of 94.6% (95% CI 93.8–95.3) and a sensitivity of 93.5% (95% CI 92.2–94.9) at the point of greatest efficiency to detect referable DR.

**Conclusions:**

A large database showed that this deep learning algorithm was accurate in detecting referable DR. This finding aids to universal healthcare systems like Brazil, optimizing screening processes and can serve as a tool for improving DR screening, making it more agile and expanding care access.

**Supplementary Information:**

The online version contains supplementary material available at 10.1186/s13098-024-01447-0.

## Background

The prevalence of type 2 diabetes has been increasing steadily in recent years and, by 2021, about 537 million people are estimated to have this condition [1] and by 2050, more than 1.31 billion (1.22–1.39) people are projected to have diabetes [2]. In Brazil, 12% of the population is diagnosed with diabetes [1, 3], making it the 6th country with the highest number of adults with diabetes in the world [1]. Diabetes is associated with microvascular and macrovascular complications, among which diabetic retinopathy (DR) stands out [4]. DR is the leading cause of blindness in working-age individuals, with a prevalence in people with diabetes of 22.2% worldwide [5] and 36.3% in Brazil [6], which may contribute to reduced quality of life depending on the severity of the disease [7].

The retina is the deepest layer of texture covering the rear of the eye, recorded by color fundus photographs (CFPs) or fundoscopy. Vessel detection and segmentation are essential in DR diagnosis [8], since the initial changes are asymptomatic [9]. Although early diagnosis and therapy can prevent severe vision loss in 90% of cases, only a small part of these patients are screened at the recommended frequency [10]. In Brazil, a study with patients with type 2 diabetes showed that only 11.5% of individuals treated under the Family Health Program, 14.9% treated in basic health units, and 35% in tertiary care had undergone fundoscopy in the last year [11]. On the other hand, another recently published study showed a screening rate of 63.9% in patients with type 1 diabetes [12]. The manual method of diagnosing DR is performed by an ophthalmologist who examines the human retinal fundus image. This manual process is very consuming in terms of the time and experience of an expert ophthalmologist, which makes developing an automated method to aid in the diagnosis of DR an essential and urgent need [13]. The lack of ophthalmologists to serve the number of patients who need to be screened is a limitation to this process [14].

Artificial intelligence is transforming ophthalmology and has been leveraged for CFP to accomplish core tasks including segmentation, classification, prediction and seem to be an alternative to solve this problem [15, 16], since the method has been tested on other populations and has shown good accuracy [17–20]. Combined with remote retinography, algorithm-based screening improves the process, selecting only higher-risk individuals to in person evaluation with an ophthalmologist [21, 22]. The deep learning system developed by Abramoff et al. has obtained a US Food and Drug Administration approval for the diagnosis of DR. It was evaluated in a prospective, although observational setting, achieving 87.2% sensitivity and 90.7% specificity [23].

Training these algorithms with diverse populations and large datasets is important to avoid biases, since previous studies suggest that the contrast between the retinal fundus and DR lesions can vary considerably between different ethnicities [24, 25]. Also, reproducibility (in different populations) is central to increasing the confidence in the scientific findings [26]. In Brazil, few studies evaluated the use of artificial intelligence to diagnose DR [27, 28].

The aim of this study is to train and evaluate the diagnostic performance of an algorithm, compared to the gold standard (evaluation by an ophthalmologist), in DR screening, using a large dataset of CFPs of Brazilian subjects, ensuring that the nuances and characteristics of this specific population are adequately captured.

## Methods

### Datasets

This study sample combined prospective and retrospective data from individuals from three public and academic institutions who applied for a CFP. A convenience sample with consecutive patients was used. Each dataset has individual characteristics, as follows:

- Endocrinology Unit of a tertiary public hospital in Porto Alegre (HCPA – Hospital de Clínicas de Porto Alegre): data from 2019 to 2021 were collected in the specialized hospital ambulatory setting prospectively and included patients more than seven years-old. The camera used was Canon CR-2 (Canon Inc., Melville, NY, USA) and two images of the posterior segment of each eye - one centered on the macula and the other centered on the disc (45° field of view) - after mydriasis induced by tropicamide 1% eye drops were captured.
- TeleOftalmo project from Rio Grande do Sul combines the state’s health authority and the Federal University telehealth initiative (TelessaúdeRS-UFRGS) and provides remote ophthalmological care to primary care patients [29, 30]. Retrospective data from individuals older than eight years-old with and without diabetes were collected from seven cities between 2020 and 2021. The camera used was ZEISS Visucam (Oberkocjen, Germany) and after patients with diabetes underwent mydriasis with tropicamide 1% eye drops, posterior segment images (30° photo centered on the disc and 45° photo centered on the macula) and photos of the nasal, temporal, superior, and inferior sector were captured.
- Ophthalmology Unit of the specialized ambulatory service from Federal University of São Paulo (UNIFESP) provides retrospective data from patients older than 10 years with diabetes between 2010 and 2020 [31]. The camera used was Canon CR-2 (Canon Inc., Melville, NY, USA) and Nikon NF-505 (Tokyo, Japan) and an image of posterior segment of each eye centered on the macula with mydriasis induced by tropicamide 0.5% was captured.

At all three sites, the images were collected by a nurse technician or researcher who had been trained in the procedure.

The study protocol was approved by the institutional Research Ethics Committees (2019 − 0113) and conducted in accordance with the Declaration of Helsinki. Data from all sets were de-identified and were in full compliance with the General Data Protection Law and local regulations.

The study is reported according to the Standards for Reporting of Diagnostic Accuracy Studies (STARD) [32] [see Additional file 1].

### Grading

After anonymization, all images were assessed for quality by the ophthalmologists and the images with interference factors, such as overexposure, inadequate focus, insufficient lighting, and excessive artifacts, were discarded. In the tertiary hospital of Porto Alegre in 2019 and TeleOftalmo, before the images were analyzed by the ophthalmologists, the EyeQ algorithm [33], which performs retinal image quality assessment, was used to select only images of sufficient quality.

The CFPs were classified into the following five categories: no retinopathy, mild non-proliferative retinopathy, moderate non-proliferative retinopathy, severe non-proliferative retinopathy, and proliferative retinopathy or macular edema, according to the International Clinical Diabetic Retinopathy (ICDR) scale [34]. There are many DR classifications applied in distinct countries and screening programs, with the ICDR scale as the most applied in open-access ophthalmological datasets [35].

The classification of DR was done by imaging. No information other than eye images was available to the evaluator. This classification was performed independently by two ophthalmologists (reference standard). Clinical information and reference standard results were not available to the assessors of the reference standard. After grading, the images were divided into two groups, non-referable DR (no DR; mild non-proliferative DR and no macular edema or clinically insignificant macular edema) and referable DR (moderate non-proliferative DR; severe non-proliferative DR; proliferative DR and/or clinically significant macular edema). The kappa value for agreement between two ophthalmologists for referable DR and non-referable DR was 0.813 (near perfect). All disagreements were adjudicated by a third grader.

### Neural network model

The goal of the neural network was to produce a binary prediction for each image: non-referable and referable DR.

For initial access to the feasibility of using a machine learning algorithm (index test) as a screening tool, 10 neural networks were trained. The codebase was inspired by Voets et al. [36] and the initial model was previously described by this study group [37]. The model used the Inception v3 architecture as a pre-trained model and was adapted and fine-tuned for our datasets. This approach leverages the knowledge encoded in the pre-trained model to solve a different but related problem more efficiently than training a new model from scratch. The same neural network architecture was used as in the original study by Gulshan et al. [15] and its replication by Voets et al. [36]. This consistency allows for a direct comparison with previous studies, highlighting that the primary differences lie in the data used and the population studied. The network was initialized with weights from the ImageNet dataset for all layers [38], except for the fully connected layer on top, which received training. After loading the weights, the fully connected dense layer with two units was added using the Sigmoid activation function [39].

Adam optimizer [40] was used for learning network weights during the training process. The initial value of the learning rate was 0.01 and the end value was 0.0037. The binary cross-entropy function was used as the loss function to estimate the logarithmic loss between the actual and predicted labels.

All images were processed in line with Voets [36], locating the center and radius of the eye fundus and resizing each image to 299 × 299 pixels, with the center of the fundus in the middle of the image. This resizing was done to match the default input size for the Inception V3 model. While larger image dimensions could be used, this would significantly increase training time. This resizing is particularly relevant for high resolution images, as compressing them to 299 × 299 pixels may result in some loss of details. However, this trade-off is necessary to maintain compatibility with the pre-trained Inception V3 architecture and to ensure efficient training. The algorithm was trained on each set for 200 epochs with batch size of 8. The datasets of the tertiary hospital in Porto Alegre, UNIFESP, and TeleOftalmo were used. The model was trained with each dataset separately and with grouped datasets into a single set. Each dataset was divided into two subsets—training and testing—in a stratified 70%/30% ratio.

Clinical information and reference standard results were not available to the performers/readers of the index test.

### Statistical analysis

The distribution of demographic and clinical characteristics was presented using mean ± standard deviation (SD) for continuous variables and by number and percentage for categorical data. For referable/non-referable DR classification, we reported the area under the receiver operating characteristic (ROC) curve, sensitivity and specificity. The sensitivity was calculated as the number of correctly predicted positive examples divided by the total number positive examples. The specificity was calculated as the number of correctly predicted negative examples divided by the total number negative examples. The performance of the algorithm in detecting referable/non-referable DR was measured by the area under the ROC curve generated by plotting sensitivity versus 1 *minus* specificity. Based on the two operating points, 2 × 2 tables were developed to characterize the sensitivity and specificity of the algorithm in relation to the gold standard which was defined as the majority decision of the ophthalmologists’ readings. The 95% confidence intervals were calculated. Three operational cutoff points were used to evaluate the performance of the algorithm: with high specificity, with greater sensitivity and maximum gain point. We excluded from analysis uninterpretable images due to poor quality. We use mean-value imputation to address missing data (age) in baseline characteristics. This study used a convenience sample, with no sample calculation conducted. Statistical significance was set at *P* < .05.

## Results

**Fig.1:**
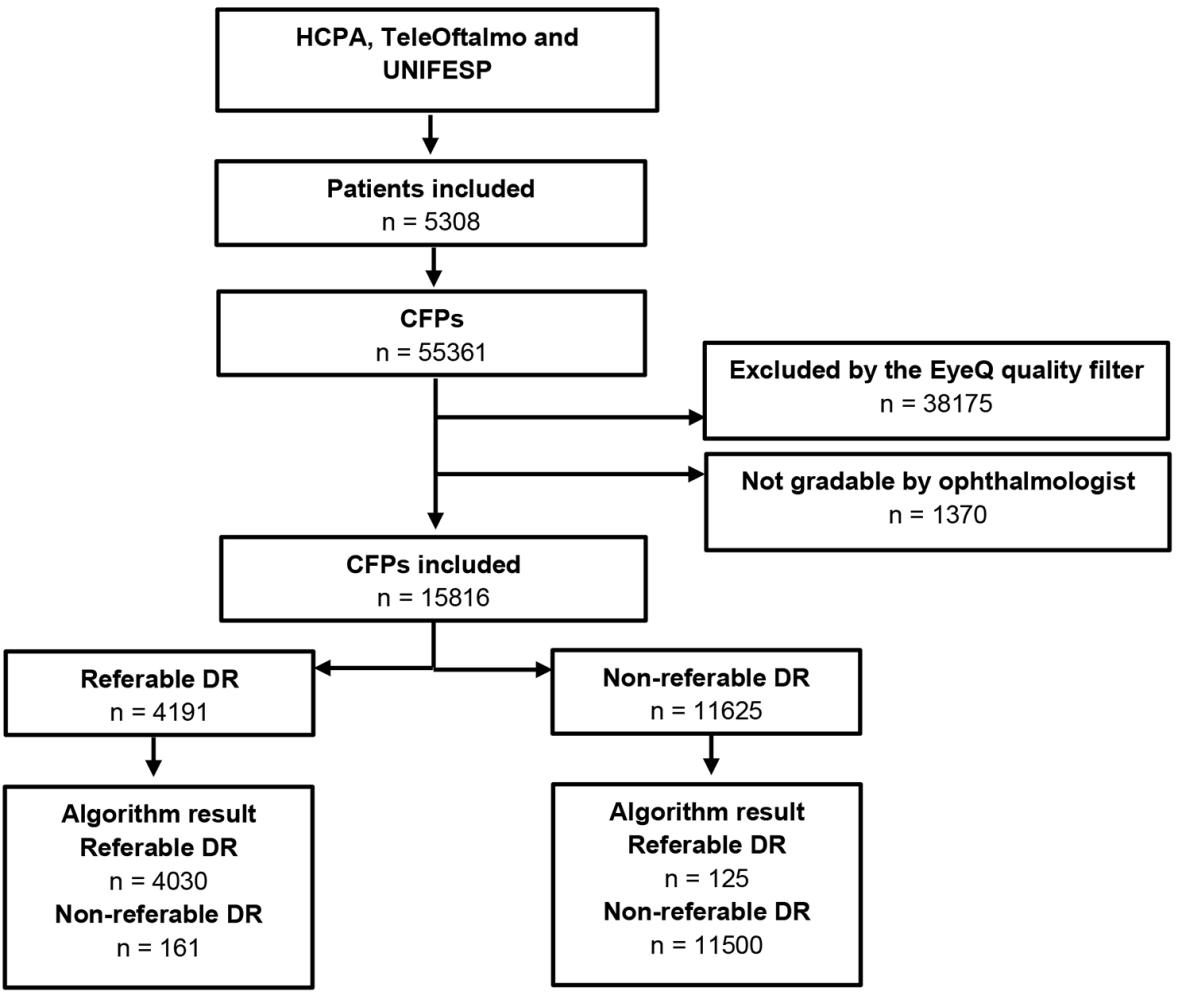
Standards for reporting of diagnostic accuracy studies (STARD) diagram for the algorithm output: referable diabetic retinopathy. DR: Diabetic Retinopathy; HCPA: Hospital de Clínicas de Porto Alegre; UNIFESP: Federal University of São Paulo; CFPs: Color Fundus Photographs

We used images of 5308 individuals and included 4590 patients (15816 images) in the analysis after quality evaluation, of which 4191 (26.5%) were classified as referable. Figure 1 shows the flow of participants during the study.

Table 1 presents the characteristics of the gradable images by dataset and by grouped dataset. In grouped dataset, the median age was 60 (7–97) years, 9684 (61.2%) were women and 4191 (26.5%) of the CFPs presented referable DR.

**Table 1:** Baseline characteristics

	Tertiary hospital in Porto Alegre 2019	Tertiary hospital in Porto Alegre 2021	TeleOftalmo	UNIFESP	All
**Patients**	651	412	2993	1252	5308
**Total CFPs**	3636	1857	47,299	2579	55,371
**Included CFPs**	2522 (69.3)	1555 (83.7)	9160 (19.3)	2579 (100)	15,816 (28.5)
**Age (years-old) among included images**	56 (12–97)	56 (7–87)	60 (8–96)	64 (10–92)	60 (7–97)
**Women among included images**	1556 (61.7)	905 (58.2)	5655 (61.7)	1568 (60.8)	9684 (61.2)
**Disease severity distribution classified by majority decision of ophthalmologists (reference standard) in included CFPs**					
**No DR**	1573 (62.4)	975 (62.7)	5651 (61.7)	1922 (74.5)	10,121 (63.9)
**Mild non-proliferative DR**	328 (13.0)	177 (11.4)	999 (10.9)	107 (4.1)	1611 (10.9)
**Moderate non-proliferative DR**	373 (14.8)	221 (14.2)	1993 (21.7)	181 (7.0)	2768 (17.5)
**Severe non-proliferative DR**	52 (2.0)	51 (3.3)	185 (2.0)	127 (4.9)	415 (2.6)
**Proliferative DR**	196 (7.8)	131 (8.4)	332 (3.6)	242 (9.4)	901 (5.7)
**Clinically significant edema**	153 (6.0)	119 (7.6)	965 (10.5)	275 (10.6)	1512 (9.5)
**Referable DR**	641 (25.4)	422 (27.1)	2578 (28.1)	550 (21.3)	4191 (26.5)

DR: diabetic retinopathy; CFPs: color fundus photographs; the data are presented as n or n (%)

The set of images of the tertiary hospital in Porto Alegre in 2019 included 651 patients and 3626 CFPs [2522 fully gradable; 641 (25.4%) referable]. The set of the same hospital in 2021 had 412 patients and 1857 CFPs [1555 fully gradable; 422 (27.1%) referable]. The TeleOftalmo set included 2993 patients and 47,299 CFPs [9160 fully gradable; 2578 (28.1%) referable], and the UNIFESP set had 1252 patients and 2579 CFPs [2579 fully gradable; 550 (21.3%) referable].

**Fig. 2:**
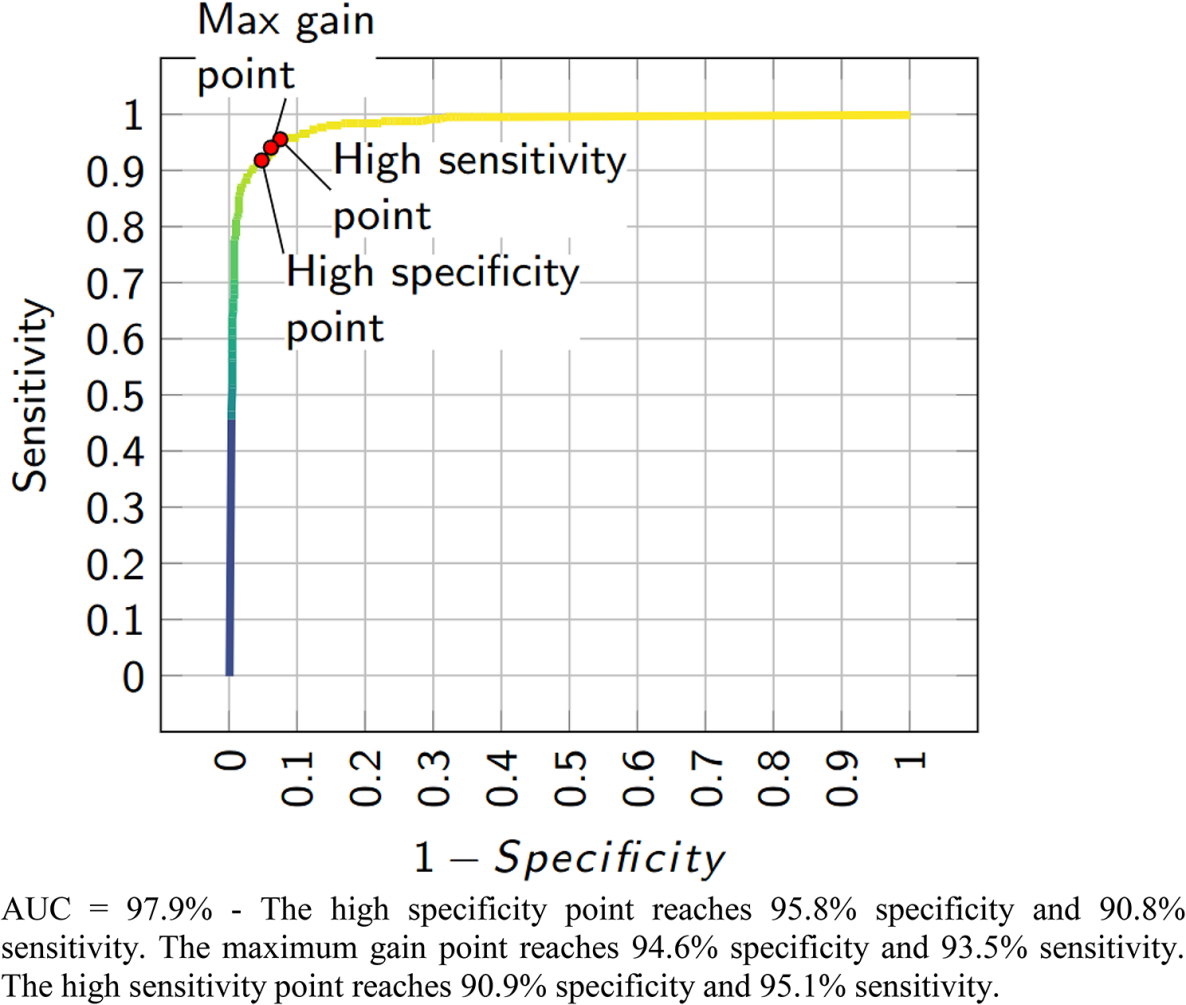
Performance of the algorithm in detecting referable diabetic retinopathy for the gradable images of all sets

Figure 2 shows the performance of the algorithm in detecting referable DR for the gradable images of all sets. Using the operational cut-off point with high specificity, the sensitivity was 90.8% (95% CI 89.2–92.4), and the specificity was 95.8% (95% CI 95.2–96.5). The point with the highest sensitivity, showing an output that would be used for a screening tool, had a sensitivity of 95.1% (95% CI 93.9–96.3) and specificity of 90.9% (95% CI 89.9–91.8). The maximum gain point had a specificity of 94.6% (95% CI 93.8–95.3) and a sensitivity of 93.5% (95% CI 92.2–94.9). Additional files 2–5 show the performance of the algorithm in detecting referable DR for the gradable images in each set evaluated.

Table 2 presents the ROC curve, sensitivity, and specificity of each image set evaluated. The algorithm achieved an area under the ROC curve of 0.98 (0.97–0.98) in grouped dataset.

No significant adverse events occurred from performing the index test or the reference standard.

**Table 2:** Area under the ROC curve, sensitivity, and specificity of each image set evaluated

	Tertiary hospital in Porto Alegre 2019	Tertiary hospital in Porto Alegre 2021	TeleOftalmo	UNIFESP	All
**Area under the ROC curve**,** (95% CI)**	0.92 (0.89–0.95)	0.95 (0.92–0.97)	0.98 (0.98–0.99)	0.94 (0.92–0.97)	0.98 (0.97–0.98)
**Sensitivity (%) (95% CI)**	95.3 (92.4–98.3)	95.3 (91.7–99.0)	95.2 (93.7–96.7)	95.3 (92.0–98.5)	95.1 (93.9–96.3)
**Specificity (%) (95% CI)**	56.7 (52.7–60.8)	72.4 (67.7–77.2)	92.6 (91.5–93.7)	80.5 (77.3–83.6)	90.9 (89.9–91.9)

ROC: receiver operating characteristic; data are presented as percentage and confidence intervals (CIs)

## Discussion

The present results showed excellent accuracy, with similarly high sensitivity and specificity, suggesting that machine learning may be an alternative to improve the workflow of DR screening in other settings other than those already tested. Sensitivity in diagnosing a disease is the most important metric of a screening test, thus, a screening program requires high sensitivity values (> 80%) [41]. However, having both, high sensitivity and high specificity is the best of both worlds: our study showed a sensitivity of 95.1% at the high sensitivity point and 90.8% at the high specificity point. A previous study undertaken with Brazilian individuals for the clinical validation of an artificial intelligence algorithm, for example, had reached a high sensitivity (97.8%) but a lower specificity (61.4%) [28]. Another study also reached high sensitivity and specificity values, but with a different methodology, as images obtained exclusively with a portable retinal camera were assessed [42]. The present study provides a comprehensive evaluation of the use of a machine learning algorithm for the detection of referable DR, making it the study in Brazil with the largest image set, the better results and with all parts of the training and validation processes performed on images of Brazilian subjects published to date.

Artificial intelligence by machine learning has become a tool that helps in reading and analyzing images [43]. Many groups around the world had previously studied the automated and semi-automated evaluation of DR [18, 28, 44–48]. DR screening is a challenge in many countries [49, 50], including Brazil, which is a continent-sized country with screening rates far below the ideal [14, 15]. The development of an algorithm with images of the Brazilian population can greatly increase the scope of DR screening, allowing only patients with changes in the initial examination to be referred to an ophthalmologist, reducing the number of referrals, so that a greater number of patients have access to specialized services, when necessary. This process, compared with human classification, can reduce screening costs when deployed as semi-autonomous screening [21, 49, 50]. In countries with structured DR screening programs, medium-term results showed a reduction in amaurosis in the population with diabetes [11].

Our study presented values close to studies performed with other populations, with a sensitivity of 95.1% and specificity of 90.9% at the high sensitivity point. The meta-analysis conducted by Wu et al. [51] showed robust performance in detecting different categories of DR, with a pooled sensitivity of 93–97% and a pooled specificity of 90–98%.

When evaluated individually, the sets of the tertiary hospital in Porto Alegre in 2019 and 2021 and UNIFESP presented lower specificity in the performance of the algorithm compared with TeleOftalmo, probably because the TeleOftalmo set is larger and the algorithm may be better fitted to its population. Studies evaluating the performance of deep learning have shown that the initial dataset for algorithm development should be large and have diverse training data in terms of patient demographics and ethnicity, image acquisition methods, and image quality [44], which is exactly what we did in the present study.

Our image set included populations from different Brazilian states, different regions of Rio Grande do Sul, and different types of cameras for performing CFP with different imaging characteristics, which was a strength of this study. These characteristics increase the external validity of our results and qualify them for future use in screening strategies for this important complication of diabetes at the national level.

This study has limitations. First, the ophthalmologists classified macular edema considering the presence of hard exudates, microaneurysms, or hemorrhage in the macular region, and studies have shown that optical coherence tomography can detect earlier changes in the vascular and retinal morphology, making it an important tool for the management and follow-up of retinal diseases, such as macular edema [52, 53]. However, the high cost of this method (equipment and human workforce) makes its use unfeasible in a continental and low/middle-income country such as Brazil. Second, the clinical information of patients was limited, which hinders further analysis of the population and its associations with the findings presented. Third, we included only images with good quality in the training and testing set. Evaluating the performance of the algorithm in a real-life study, without selecting good quality images, is necessary.

## Conclusion

This study showed that deep machine learning algorithm is reproducible in a diverse population, with elevated sensitivity and specificity for the detection of referable DR. This classification is useful in universal healthcare systems (such as the Brazilian example) as a tool to improve the screening flow of DR in the country. Further research is needed to determine the real-life applicability of the algorithm, its cost-effectiveness to assess whether it can improve the care of patients with diabetes, and the regulation of its use on a large scale.

### Electronic supplementary material

Below is the link to the electronic supplementary material.

Supplementary Material 1

Supplementary Material 2

Supplementary Material 3

Supplementary Material 4

Supplementary Material 5

## Data Availability

The study dataset is available from the corresponding author upon reasonable request.

## Abbreviations

CFP: Color fundus photograph
CIs: Confidence intervals
DR: Diabetic retinopathy
UNIFESP: Federal University of São Paulo
HCPA: Hospital de Clínicas de Porto Alegre
ICDR: International Clinical Diabetic Retinopathy
ROC: Receiver operating characteristic
SD: Standard deviation
STARD: Standards for Reporting of Diagnostic Accuracy Studies
