# Supplementary material for: Advancing healthcare with artificial intelligence: diagnostic accuracy of machine learning algorithm in diagnosis of diabetic retinopathy in the Brazilian population
Source: Diabetol Metab Syndr. 2024 Aug 29;16:209. doi: 10.1186/s13098-024-01447-0 (PMC11360296; doi:10.1186/s13098-024-01447-0)
Supplement: Supplementary file 4 — Supplementary Material 4 [file 13098_2024_1447_MOESM4_ESM.docx]

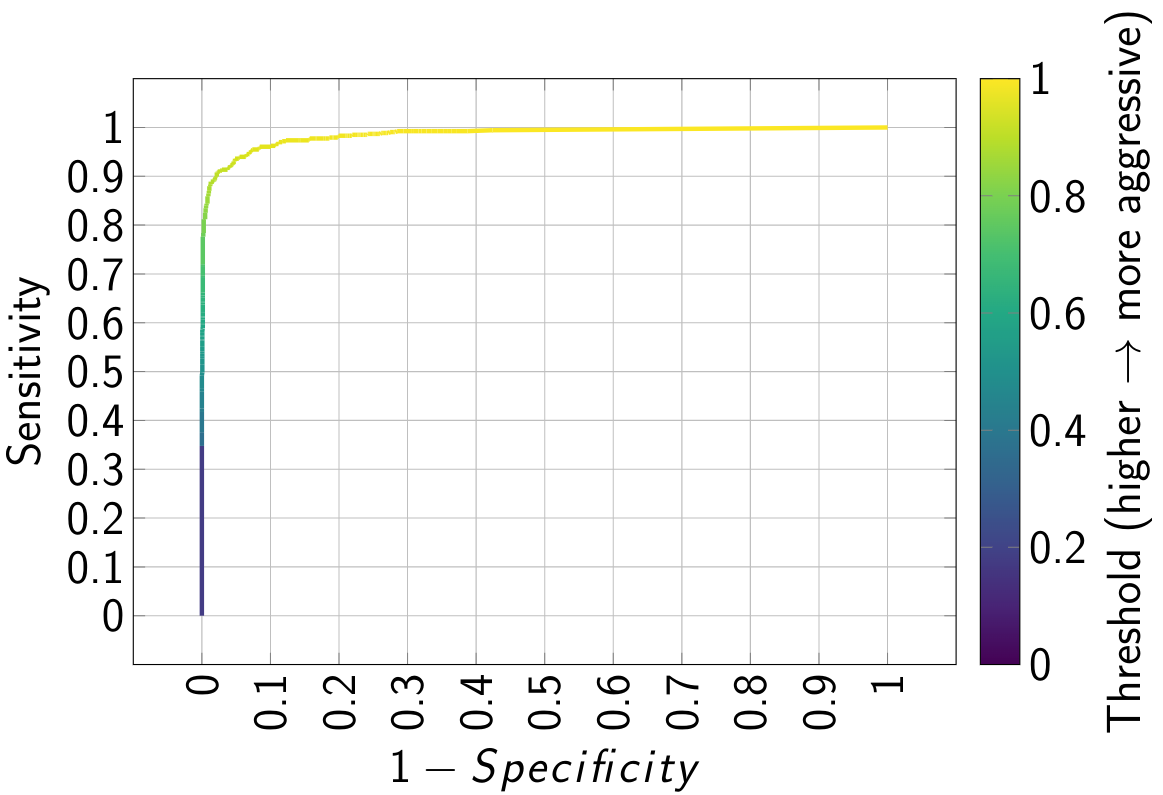


﻿AUC = 98.6% - The high specificity point reaches 96.0% specificity and 91.7% sensitivity. The maximum gain point reaches 95.1% specificity and 93.6% sensitivity. The high sensitivity point reaches 92.6% specificity and 95.2% sensitivity.
