# Supplementary material for: Advancing healthcare with artificial intelligence: diagnostic accuracy of machine learning algorithm in diagnosis of diabetic retinopathy in the Brazilian population
Source: Diabetol Metab Syndr. 2024 Aug 29;16:209. doi: 10.1186/s13098-024-01447-0 (PMC11360296; doi:10.1186/s13098-024-01447-0)
Supplement: Supplementary file 5 — Supplementary Material 5 [file 13098_2024_1447_MOESM5_ESM.docx]

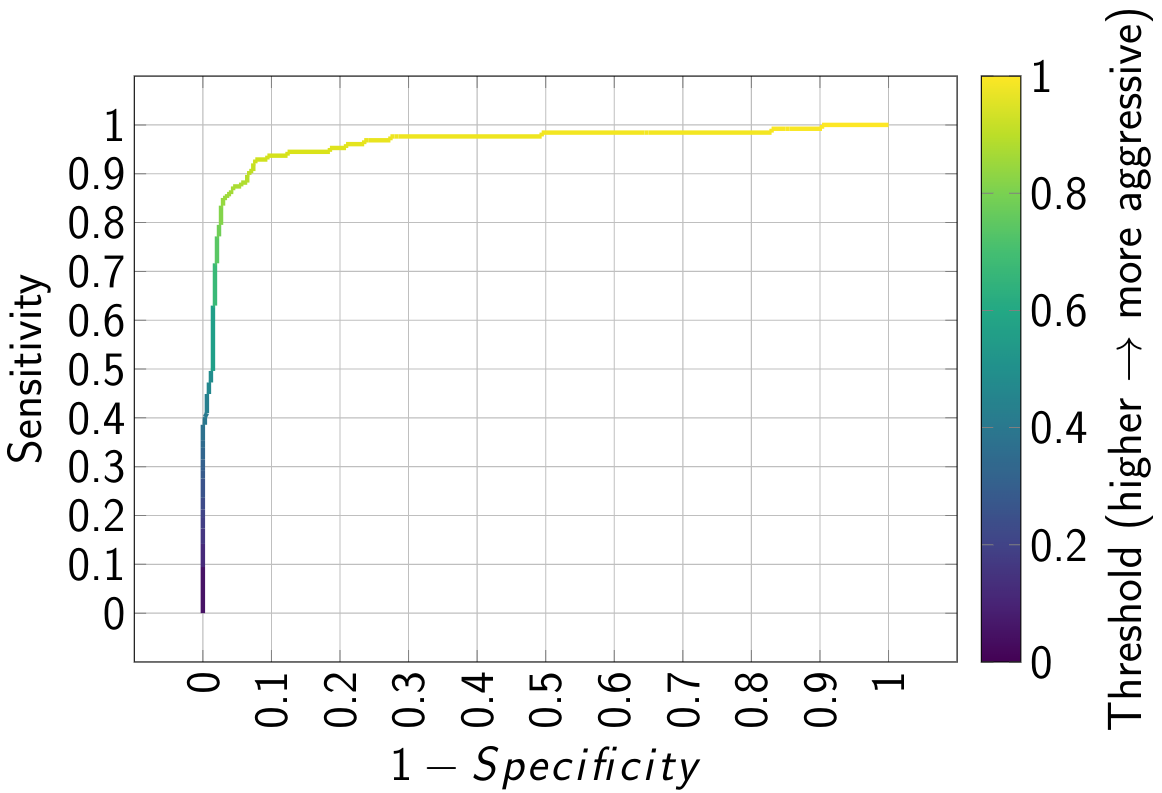


﻿AUC = 95.5% - The high specificity point reaches 95.6% specificity and 76.3% sensitivity. The maximum gain point reaches 87.0% specificity and 96.0% sensitivity. The high sensitivity point reaches 80.5% specificity and 95.2% sensitivity.
